# Survival outcomes of stage I colorectal cancer: development and validation of the ACEPLY model using two prospective cohorts

**DOI:** 10.1186/s12916-022-02693-7

**Published:** 2023-01-04

**Authors:** Qingbin Wu, Pengju Chen, Chi Shu, Lin Chen, Zechuan Jin, Jun Huang, Xin Wang, Xue Li, Mingtian Wei, Tinghan Yang, Xiangbing Deng, Aiwen Wu, Yazhou He, Ziqiang Wang

**Affiliations:** 1 Colorectal Cancer Center, Department of General Surgery, West China Hospital, Sichuan University, Chengdu, China; 2 West China School of Public Health and West China Fourth Hospital, Sichuan University, Chengdu, China; 3 Key Laboratory of Carcinogenesis and Translational Research (Ministry of Education), Department of Unit III & Ostomy Service, Gastrointestinal Cancer Centre, Peking University Cancer Hospital & Institute, Beijing, China; 4 Department of Vascular Surgery, West China Hospital, West China School of Medicine, Sichuan University, Chengdu, China; 5 Key Laboratory of Ministry of Education for Gastrointestinal Cancer, Fujian Medical University, Fuzhou, China; 6 Department of Epidemiology and Medical Statistics, West China School of Public Health and West China Fourth Hospital, Sichuan University, Chengdu, China; 7 Department of Big Data in Health Science, School of Public Health, Center of Clinical Big Data and Analytics of The Second Affiliated Hospital, Zhejiang University School of Medicine, Hangzhou, China; 8 Centre for Global Health, Usher Institute, The University of Edinburgh, Edinburgh, UK; 9 Department of Oncology, West China School of Public Health and West China Fourth Hospital, Sichuan University, Chengdu, China

**Keywords:** Colorectal cancer, Early stage, Prognosis, Risk factor, Prediction model

## Abstract

**Background:**

Approximately 10% of stage I colorectal cancer (CRC) patients experience unfavorable clinical outcomes after surgery. However, little is known about the subset of stage I patients who are predisposed to high risk of recurrence or death. Previous evidence was limited by small sample sizes and lack of validation.

**Methods:**

We aimed to identify early indicators and develop a risk stratification model to inform prognosis of stage I patients by employing two large prospective cohorts. Prognostic factors for stage II tumors, including T stage, number of nodes examined, preoperative carcinoma embryonic antigen (CEA), lymphovascular invasion, perineural invasion (PNI), and tumor grade were investigated in the discovery cohort, and significant findings were further validated in the other cohort. We adopted disease-free survival (DFS) as the primary outcome for maximum statistical power and recurrence rate and overall survival (OS) as secondary outcomes. Hazard ratios (HRs) were estimated from Cox proportional hazard models, which were subsequently utilized to develop a multivariable model to predict DFS. Predictive performance was assessed in relation to discrimination, calibration and net benefit.

**Results:**

A total of 728 and 413 patients were included for discovery and validation. Overall, 6.7% and 4.1% of the patients developed recurrences during follow-up. We identified consistent significant effects of PNI and higher preoperative CEA on inferior DFS in both the discovery (PNI: HR = 4.26, 95% CI: 1.70–10.67, *p* = 0.002; CEA: HR = 1.46, 95% CI: 1.13–1.87, *p* = 0.003) and the validation analysis (PNI: HR = 3.31, 95% CI: 1.01–10.89, *p* = 0.049; CEA: HR = 1.58, 95% CI: 1.10–2.28, *p* = 0.014). They were also significantly associated with recurrence rate. Age at diagnosis was a prominent determinant of OS. A prediction model on DFS using *A*ge at diagnosis, *CE*A, *P*NI, and number of *LY*mph nodes examined (ACEPLY) showed significant discriminative performance (C-index: 0.69, 95% CI:0.60–0.77) in the external validation cohort. Decision curve analysis demonstrated added clinical benefit of applying the model for risk stratification.

**Conclusions:**

PNI and preoperative CEA are useful indicators for inferior survival outcomes of stage I CRC. Identification of stage I patients at high risk of recurrence is feasible using the ACEPLY model, although the predictive performance is yet to be improved.

**Supplementary Information:**

The online version contains supplementary material available at 10.1186/s12916-022-02693-7.

## Background

Colorectal cancer (CRC) is the second leading cause of cancer-related death worldwide in 2020 [1]. Although patients with stage I disease generally experience favorable prognosis, there were reportedly 10% of them who developed recurrent tumors within 5 years after curative resection [2–7]. With strengthened population-based screening programs, a continuing increase in the proportion of stage I cases is expected [8], underpinning compelling rationale to identify stage I patients who are predisposed to inferior survival outcomes.

Our previous umbrella review found a dearth of solid evidence on factors influencing survival outcomes nor available prediction tools for stage I CRC [9]. The major challenge lies in the low frequency of unfavorable outcome events as well as the lack of prior knowledge on candidate factors. Meanwhile, well-established prognostic indicators for stage II tumors to inform adjuvant treatment, such as suboptimal lymph node retrieval and perineural invasion (PNI), have been recommended by guidelines from international societies, such as the European Society of Medical Oncology (ESMO) and National Comprehensive Cancer Network (NCCN) [10, 11]. Given that most of these factors are also present in stage I CRC, they may also exert impact on survival outcomes of these patients. Herein, we report a discovery-validation study investigating prognostic effects of these factors and developing a prognostication tool for stage I patients using two prospective cohorts.

## Methods

### Study population

This study enrolled consecutive patients diagnosed with CRC at the West China Hospital (WCH, Chengdu, China) between April 2009 to April 2016 as the discovery cohort, whereas the validation cohort was comprised of individuals diagnosed within the same time span at the Peking University Cancer Hospital & Institute (PUCHI, Beijing, China). The study was performed in accordance with the Declaration of Helsinki and reported adhering to the STROBE statement for observational studies [12], with the STROBE checklist presented in Additional file 1.

We included patients who underwent R0 resection with pathologically diagnosed stage I tumor according to the American Joint Committee on Cancer (AJCC) TNM staging manual. The exclusion criteria included the following: (1) patients having received any neoadjuvant treatment, (2) patients with multiple tumors, (3) patients undergoing endoscopic resection due to unavailable pathological evidence of lymph node status, and (4) patients who died within one month after surgery. Of note, patients having received endoscopic resections prior to the curative surgery were also enrolled as evidence showed that the preceding endoscopic procedure had no significant adverse effects on long-term survival outcomes (5).

### Candidate prognostic factors

Based on the recommendations from NCCN, prognostic parameters for high-risk stage II colon cancer patients included: pathologically confirmed T4 stage, undifferentiated or poorly differentiated tumors (G3 or G4), lymphovascular invasion (LVI), PNI, lymph nodes sampling < 12, with obstruction or perforation, and positive resection margins [11, 13]. These factors above were also listed in the recommendations from the Chinese Society of Clinical Oncology (CSCO) [14]. In addition, the ESMO guideline noted the level of preoperative carcinoma embryonic antigen (CEA) as a prognostic factor for stage II tumors [10]. As positive margins and presentations of obstruction or perforation rarely occurred in stage I patients, we included the remaining six factors, namely T stage (T2 vs. T1), tumor grade (G3 or G4 vs G1 or G2), LVI, PNI, suboptimal lymph node examination (< 12), and CEA, in conjunction with age and gender as candidate covariates.

### Patient follow-up and survival outcomes

A standard follow-up scheme was applied to participants in both cohorts, and details can be found in Additional file 2. In view of the low prevalence of inferior outcomes, we adopted disease-free survival (DFS) as the primary outcome to obtain maximum statistical power. The DFS was defined as the time span from the date of surgery to recurrence at any sites, death, or the date of last follow-up. Recurrence was confirmed by biopsy or diagnosed by at least two radiologists via CT or MRI scans. We also employed overall survival (OS) and recurrence rate as secondary outcomes. CRC-specific deaths and site-specific recurrences were also investigated as additional outcomes for sensitivity analysis. The latest patient follow-up for both cohorts was completed in May 2020.

### Statistical analysis

#### Identification of prognostic indicators

In descriptive analysis, log-transformation was conducted to normalize the continuous variables with skewed distribution. In consideration of power loss and possible biases caused by dichotomization, we kept the original scale of continuous variables in survival analysis [15]. With respect to missing data, we adopted a multiple imputation approach to impute the missing values of patient characteristics [16]. Survival rates were estimated using the Kaplan–Meier approach. In discovery analysis, we first fitted univariable proportional hazard Cox models to estimate effects of candidate risk factors on DFS and other outcomes. A two-sided *p* value < 0.05 was used as the threshold to select potential factors, which were then validated the using the PUCHI cohort. A successful validation was determined by a two-sided *p* < 0.05.

#### Predictive modelling and visualization

A multivariable Cox model including features identified from univariable analysis was fitted in the WCH cohort, factors with significant impact (*p* < 0.05) in this model were selected, and their coefficients were retained to generate predicted survival estimates in the validation cohort in order to evaluate the external model performance. We conducted analysis of variance to examine non-linearity of continuous predictors, and restricted cubic splines (RCS) were utilized to model any non-linear associations [17]. We then calculated a concordance index (C-statistic) along with the 95% confidence interval (CI) to assess the discriminative ability of the model. A C-statistic with a 95% CI excluding 0.5 indicated significant discriminative ability. We then constructed calibration curves by plotting the predictive 5-year survival estimates against the observed rates and visually assessed the discrepancies. A nomogram along with an online calculator was developed based on the validated model to provide a plug-in tool for clinical use.

To visualize the model performance, a prognostic index was created using the linear predictor, and a Kaplan–Meier curve for the validation cohort was plotted based on the optimal cut-off value derived from the discovery phase using an iterative approach via the X-tile software [18]. We also plotted the time-dependent trends of area under the curves (AUC) to exhibit discriminative ability of the prediction model at various follow-up time [19]. With respect to possible clinical utility, decision curve analysis (DCA) was conducted to estimate net benefits of applying the prediction model in clinical decision-making compared to the null model in which all participants were considered at the same risk level [20].

## Results

### Patient characteristics

**Fig. 1:**
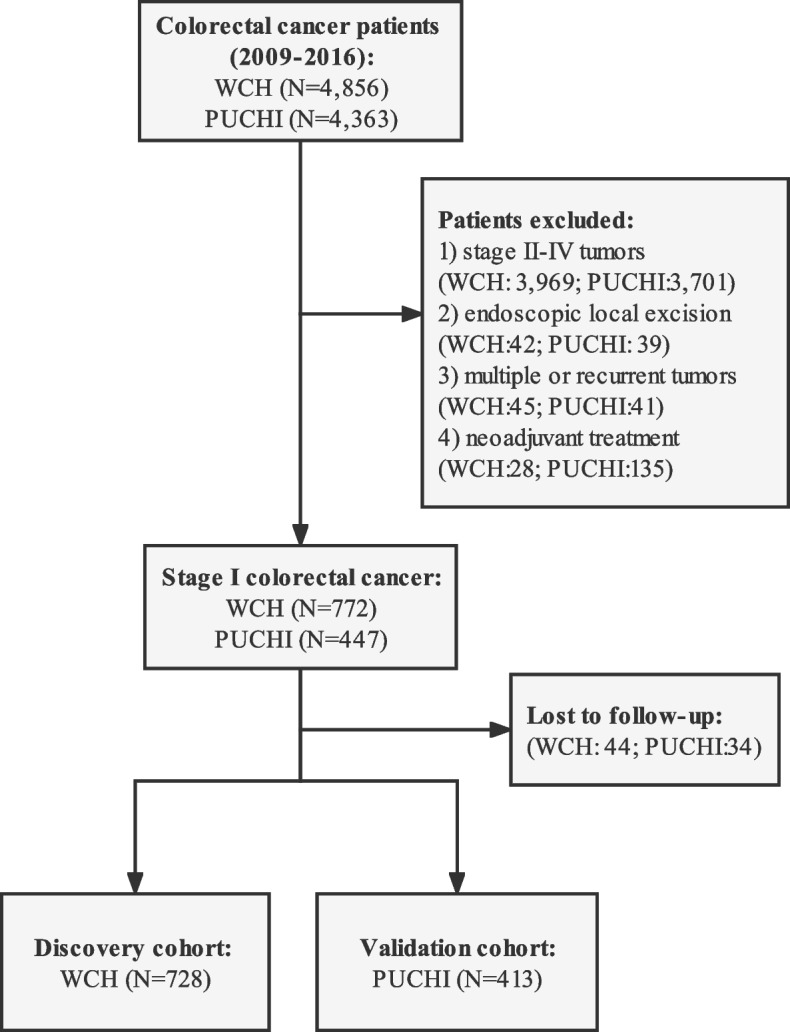
Diagram for patient selection of two study cohorts

Based on the inclusion criteria, 728 from WCH and 413 patients from PUCHI were included (diagram of patient selection in Fig. 1). Essential characteristics for enrolled patients are summarized in Table 1. There was a significantly larger proportion of rectal cancer patients and a smaller proportion of left colon cancer patients in the discovery cohort (*p* < 0.001). In addition, we observed a higher percentage of patients with poor tumor differentiation (G3 or G4) in the discovery cohort (19.7% vs. 7.3%).

**Table 1:** Summarized distribution of essential characteristics of the discovery and validation cohorts

	Discovery cohort (WCH)	Validation cohort (PUCHI)	*p*-value*
Variables	*n* = 728	*n* = 413	
*Basic characteristics*^a^			
Gender			
Male	406 (55.8%)	239 (57.9%)	0.532
Female	322 (44.2%)	174 (42.1%)	
Age (years)	62.0 (54.0–70.0)	62.0 (55.0–70.0)	0.538
Surgical procedure			
Open	519 (71.3%)	296 (71.7%)	0.946
Laparoscopic	209 (28.7%)	117 (28.3%)	
Tumor site			
Right colon	49 (6.7%)	36 (8.7%)	< 0.001
Left colon	109 (15.0%)	104 (25.2%)	
Rectum	570 (78.3%)	273 (66.1%)	
Tumor size (cm)	3.0 (2.0–3.5)	2.8 (2.0–4.0)	0.854
Follow-up (months)	62.0 (53.0–71.0)	63.0 (41.0–77.0)	0.065
*Candidate risk factors*			
CEA (ng/ml)	2.4 (1.5–3.7)	2.3 (1.4–3.7)	0.411
Grade			
G1 + G2	578 (80.3%)	383 (92.7%)	< 0.001
G3 + G4	142 (19.7%)	30 (7.3%)	
T stage			
T1	170 (23.4%)	100 (24.2%)	0.798
T2	558 (76.6%)	313 (75.8%)	
No. lymph nodes retrieval	12.0 (8.0–16.0)	12.0 (8.0–15.0)	0.995
Lymphovascular invasion	30 (4.1%)	19 (4.6%)	0.817
Perineural invasion	18 (2.5%)	15 (3.6%)	0.348

*WCH* West China Hospital, *PUCHI* Peking University Cancer Hospital & Institute, *CEA* Carcinoma embryonic antigen

^*^p values were derived from chi-square tests for categorical data and Mann–Whitney *U* tests for continuous data

^a^Characteristics were summarized using count (percentage) for categorical data and median (quartile) for continuous data

During the follow-up time span, 39 deaths along with 49 recurrences (44 distant and five local) occurred in the discovery cohort; meanwhile, 28 patients died and 17 developed recurrent tumors, among whom 13 had distant recurrences, in the validation cohort. We observed a 5-year DFS of 91% (95% CI: 89–93%) for the discovery cohort and 92% for the validation cohort (95% CI: 90–95%, log-rank test *p* = 0.950) (Additional file 3: Figure S1). Similarly, we did not find significant differences with respect to 5-year OS (95% vs. 94%, *p* = 0.330) and recurrence rate (6.9% vs. 3.8%, *p* = 0.130) across the two cohorts.

### Risk factors for survival outcomes

In discovery analysis, elder age at diagnosis and higher preoperative CEA was significantly associated with worse DFS (age per 1 year: HR = 1.04, 95% CI: 1.02–1.07, *p* < 0.001, CEA per 1 log-transformed unit: HR = 1.46, 95% CI: 1.13–1.87, *p* = 0.003). We also found significant effect of PNI on DFS (HR = 4.26, 95% CI: 1.70–10.67, *p* = 0.002). These three factors retained their significant influence on DFS in the validation cohort (details in Table 2). We failed to validate T2 stage and suboptimal lymph node examination (< 12) as prognostic indicators in validation analysis although they were significantly associated with inferior DFS in the WCH cohort (Table 2). With regard to secondary outcomes, age and CEA was observed to be linked with OS, while PNI were linked to tumor recurrence rates in both discovery and validation cohorts (*p* < 0.05, details in the Table 2).

**Table 2:** Summarized effect estimates of univariable Cox regression in discovery and validation analysis

	**Disease-free survival**							**Overall survival**						**Recurrence**				
	Discovery			Validation			Discovery			Validation			Discovery			Validation		
*Risk factors*	HR	95% CI	*p*	HR	95% CI	*p*	HR	95% CI	*p*	HR	95% CI	*p*	HR	95% CI	*p*	HR	95% CI	*p*
Age	**1.04**	**(1.02, 1.07)**	** < 0.001**	**1.05**	**(1.02, 1.08)**	**0.004**	**1.10**	**(1.07, 1.14)**	** < 0.001**	**1.05**	**(1.01, 1.09)**	**0.012**	1.01	(0.99, 1.04)	0.376	—		
Gender (male)	1.44	(0.87, 2.37)	0.156	—			1.62	(0.83, 3.14)	0.158	—			1.26	(0.71, 2.24)	0.433	—		
Grade (G3 or 4)	0.90	(0.48, 1.67)	0.730	—			1.06	(0.49, 2.30)	0.888	—			1.06	(0.53, 2.12)	0.877	—		
CEA^a^	**1.46**	**(1.13, 1.87)**	**0.003**	**1.58**	**(1.10, 2.28)**	**0.014**	**1.61**	**(1.17, 2.20)**	**0.003**	**1.89**	**(1.26, 2.83)**	**0.002**	**1.54**	**(1.16, 2.04)**	**0.003**	1.42	(0.81, 2.49)	0.215
LVI	2.06	(0.83, 5.14)	0.120	—			0.66	(0.09, 4.80)	0.680	—			2.21	(0.79, 6.14)	0.130	—		
Node retrieval (< 12)	**2.01**	**(1.22, 3.32)**	**0.006**	1.74	(0.91,3.33)	0.093	1.78	(0.94, 3.40)	0.079	—			**2.30**	**(1.26, 4.17)**	**0.006**	1.63	(0.63, 4.23)	0.316
PNI	**4.26**	**(1.70, 10.67)**	**0.002**	**3.31**	**(1.01, 10.89)**	**0.049**	**6.45**	**(2.27, 18.36)**	** < 0.001**	NA^b^			**4.34**	**(1.56, 12.13)**	**0.005**	**7.33**	**(2.07, 25.95)**	**0.002**
T stage (T2)	**2.27**	**(1.09, 4.76)**	**0.029**	1.05	(0.49, 2.22)	0.908	**3.69**	**(1.14, 11.97)**	**0.030**	1.05	(0.44, 2.47)	0.919	2.21	(0.94, 5.18)	0.070	—		

*CEA* Carcinoma embryonic antigen, *LVI* Lymphovascular invasion, *PNI* Perineural invasion, *NA* Not available

^a^CEA levels were log-transformed

^b^Effects could not be estimated due to scarce events in the risk group

As for sensitivity analysis, higher preoperative CEA was also observed to be associated with inferior CRC-specific survival (CSS) in the two cohorts (*p* < 0.05, Additional file 4: Table S1). With respect to recurrence types, risk factors presented similar distributions across patients with recurrences at local and distant sites (Additional file 4: Table S2). Given the rareness of recurrences at local sites (14%), site-specific survival analysis was only conducted for distant sites, and presence of PNI retained as significant indicator for higher risk of distant recurrences in both cohorts (*p* < 0.05, Additional file 4: Table S3).

### Prediction of inferior outcomes

**Fig. 2:**
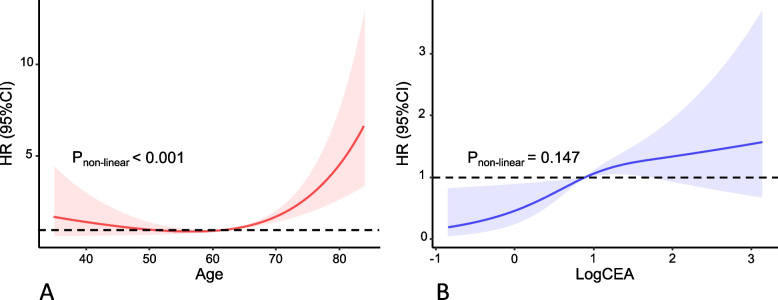
Dose–response association of age at diagnosis and preoperative CEA level with disease-free survival. **A** Non-linear relationship between age and DFS. **B** Linear relationship between log-transformed CEA and DFS

**Fig. 3:**
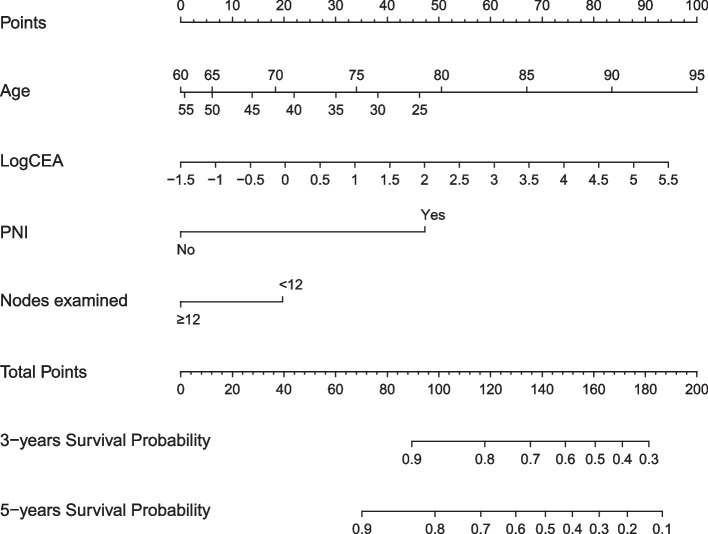
Nomogram of the ACEPLY tool predicting 3- and 5-year disease-free survival of stage I colorectal cancer patients

By fitting multivariable Cox models in the discovery set, we identified four predictors, i.e., *A*ge, *CE*A, *P*NI, and *LY*mph nodes examined (Additional file 4: Table S4, *p* < 0.05), which were subsequently utilized to develop the ACEPLY model forecasting DFS. Analysis of variance found significant non-linearity (*p* < 0.05) between age at diagnosis and DFS, and therefore, a restricted cubic spline (RCS) was applied to model the categorized effects of age. The dose–response relations between age, CEA, and DFS are shown in Fig. 2. The prediction rule is presented as a nomogram in Fig. 3. We also created a web-based ACEPLY tool to provide plug-in calculation as well as visualization of predicted DFS (https://webcalculator.shinyapps.io/DFS_ACEPLY/). For example, the ACEPLY yielded an expected 5-year DFS of 57.0% for a 65-year stage I patient with preoperative CEA of 20 ng/ml, positive PNI, and less than 12 nodes examined.

**Fig. 4:**
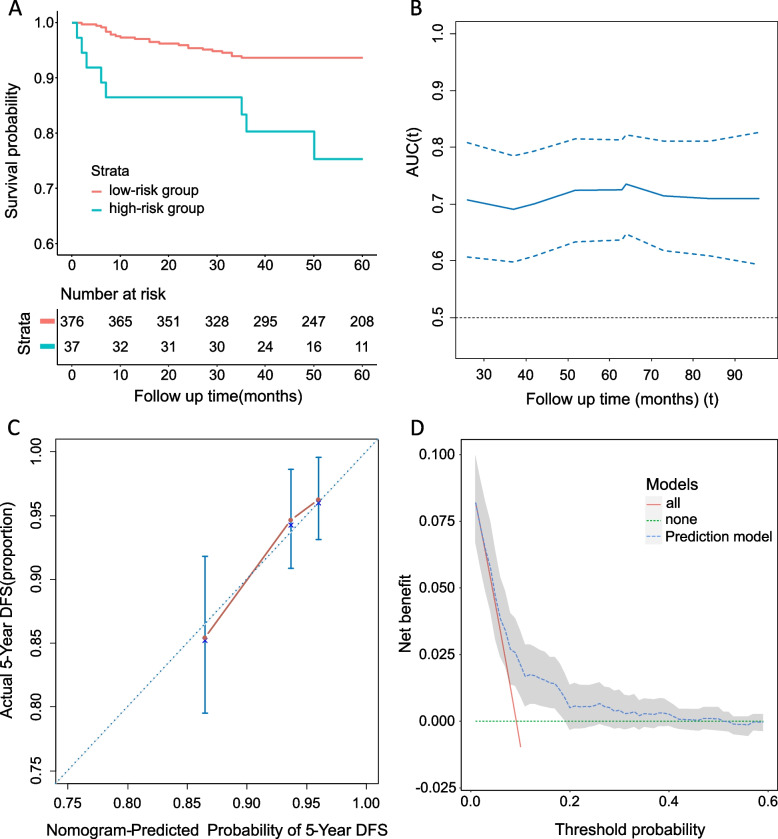
Performance of the ACEPLY model on DFS. **A** Kaplan–Meier curve of high- and low-risk group of stage I patients in the validation cohort based on linear prognostic index with a cut-off value derived from the discovery cohort. **B** Time-dependent area under the curve (AUC) of the prediction model validated in the external cohort. **C** Model calibration in the validation cohort. **D** Decision curve analysis of the prediction model. The probability threshold indicates the ratio of benefit of true positives vs. the harm of false positives

With respect to the model performance, the validation cohort was divided into high- and low-risk group based on the optimal cut-off value of the prognostic index derived from the discovery cohort. Patients in the high-risk group of the validation cohort showed significantly inferior survival outcomes (Fig. 4A). We evaluated the external discriminative performance and obtained a significant overall concordance index of 0.69 (95% CI: 0.60–0.77). This was further confirmed by time-dependent AUC analysis at various time points, with the discriminative ability peaked after 5 to 6 years since diagnosis (Fig. 4B). Acceptable calibration was observed based on the overall agreement between the predicted and observed 5-year DFS (calibration plot in Fig. 4C). DCA identified significant net benefit of adding the model to decision-making at a relatively low threshold probability (0.1–0.2, Fig. 4D). Prediction models were also developed for OS and recurrence using the same approach (effect estimates presented in Additional file 4: Table S4), and their performance is shown in Additional file 3 (Figure S2 for OS and Figure S3 for recurrence).

## Discussion

With the improvement in cancer screening, more tumors will be detected at an earlier stage, necessitating more attention to stage I patients. The current study identified and validated indicators and provided a clinically-useful prediction tool that enabled early recognition of stage I patients at higher risk of poor outcomes.

PNI presents the growth and invasion of tumor cells into nerves in the surrounding microenvironment. Previous evidence indicated that peripheral nerves, as essential components of the tumor microenvironment, can facilitate tumor progression and metastasis via the nerve sheaths [21, 22]. This resonates with our findings on the specific effect of PNI on higher risk of CRC distant recurrence. Higher prevalence of PNI has been observed in more lethal cancers (present in 90% of pancreatic cancer [23]). In the case of CRC, Liebig et al. found that PNI prevalence increased with more advanced tumor stages [24]. However, they failed to observe any PNI in 46 stage I patients, pointing to a pressing need for investigation in larger cohorts. Our multi-center study observed positive PNI in 2.5–3.6% of stage I patients. Albeit occurring less frequently, eight out of 33 (24.2%) PNI-positive patients developed unfavorable outcomes, which was even parallel to the reported DFS of stage II patients [25], indicating that PNI can provide certain clinical utility in identifying the small subset of stage I patients who are predisposed to inferior outcomes.

Preoperative serum CEA level is a well-studied biomarker for recurrence risk in stage II–III CRC [26, 27] but remains less investigated for stage I patients. An empirical cut-off of 5 ng/ml was widely used by registry-based studies [28]; however, this value has been proven suboptimal by later modeling efforts using pooled data from trials [26]. Moreover, evidence has demonstrated that dichotomization of variables in continuous scale could result in loss of statistical power and possible biased estimation [15, 29], which would be detrimental for investigation in less frequent outcome events like the current study. Thus, we modeled the relation between CEA and DFS while retaining the original continuous scale, and our findings add to current knowledge by unveiling the linear relationship, which was subsequently leveraged in strengthening the predictive performance of the ACEPLY tool.

With regard to other factors, we observed a similar, yet non-significant (*p* = 0.09) impact of lymph node sampling < 12 on DFS in validation, which could be attributed to inadequate power of the validation cohort. However, it still showed predictive performance. The ESMO guideline listed the number node sampling as a major prognostic factor for stage II disease [10]. In view of the fact the missing affected nodes is less likely in tumors at an earlier stage, the expected effect of node sampling could be smaller among stage I than stage II patients. Therefore, future large validation study is still needed to confirm the exact effect. The insufficient statistical power might also have played a role in our analysis on the effect of T2 stage and LVI, where consistent direction of effects were reported by previous studies [3, 30]. In concordance with our findings, Lee et al. did not observe significant influence of tumor grade on recurrence risk for stage I CRC [3]. This could be attributed to the strong correlation between tumor grade and TNM stage [31], which could confound the underlying effect of tumor grade.

Our study observed that elder age at diagnosis was independently associated with poor OS instead of recurrence rate. A latest population-based study across all tumor stages reported similar findings, but meanwhile, it detected a significant effect modification on age by tumor stage [32]. More importantly, a reduction in survival benefit was observed in patients of 24 years or younger compared with those from 35 to 39 [32]. Another analysis combining data from six trials suggested adverse prognostic impact of young age in stage III patients [33], highlighting the need for investigating stage-specific effect of age. Our study offered a glimpse into a potential non-linear relation between age at diagnosis and DFS of stage I CRC patients, although this finding merits further verification by future evidence.

The low prevalence of both risk factors and outcome event renders it rather challenging to develop statistical models for risk stratification among stage I patients. Given the absence of published prognostication tool [34], the ACEPLY model presented a pioneer effort in the field, more importantly, with externally validated model performance, to help clinicians inform individualized patient outcomes. Although clinical net benefit was identified, the low probability threshold pointed to escalated odds of false positive predictions, and this caveat needs to be fully considered before any adjuvant treatment or more intensive follow-up scheme being adopted. In addition, the prediction accuracy is yet to be further improved with more risk factors being discovered and added into the current model.

As opposed to the European population [35], rectal cancer tends to be more dominant in eastern Asia. In accordance with our results, a reported 50 ~ 80% of CRC patients presented rectal tumors in China [30, 36]. Similarly, rectal cancer had the highest incidence among all sites along the large bowel in South Korea [37]. This might be attributed to distinct genetic background and dietary style in the area [37]. Rectal cancer has been reportedly enriched particularly in an early stage. The US national cancer registry identified a significantly greater percentage of rectal cancer among stage 0 or I CRC patients than stage II (35% vs. 24%) [38]. More prominent symptoms, such as bleeding, might render tumors in the rectum easier to be detected at an earlier stage. Our cohorts also featured a large proportion of open resections in line with the dominance of rectal tumors. Although tumor site and surgical approach had no significant impact on survival outcomes in our study as well as other previous reports[39, 40], our findings including the ACEPLY tool merit re-calibration when applied to other populations with varied structures of these covariates.

Although this is, to our knowledge, the largest study with independent validation targeting survival outcomes of stage I CRC, the sample size is still the major limitation of the current study. The relatively rare events, such as PNI and tumor recurrences, hindered more extensive investigations in possible factors and further subgroup analysis (e.g. by recurrence subtypes) given the grossly risen type I error due to multiple testings. A second limitation is that our validation cohort is overall smaller in sample size than the discovery cohort, leading to inadequate statistical power to replicate potential discoveries, such as the impact of suboptimal lymph node examination. Last but not the least, our study featured the local patient population, for example the high proportion of rectal cancer, and thus, our findings including the prediction tool, merit further external validation and re-calibration before applied to other populations.

## Conclusions

In conclusion, the present study discovered and validated the utility of PNI and preoperative CEA in prognostication of stage I CRC. Moreover, an externally validated prediction tool was developed for clinical use to identify stage I CRC patients at high-risk for inferior survival outcomes. Future collaborative efforts are warranted to aggregate larger patient cohorts with the hope of revealing more prognostic factors to further improve prediction accuracy.

## Supplementary Information

**Additional file 1.** STROBE checklist for reporting observational studies.

**Additional file 2.** Follow-up scheme for patients.

**Additional file 3:** **Figure S1-S3.** **Figure S1.** Kaplan-Meier curves of DFS (A), OS (B) and recurrence rate (C) for the two study cohorts. **Figure S2.** Performance of the multivariable prediction model on OS. **Figure S3.** Performance of the multivariable prediction model on recurrence rate.

**Additional file 4:** **Table S1-S4.** **Table S1.** Summarized results of sensitivity analysis of associations between candidate risk factors and CRC-specific survival. **Table S2.** Summarized results of sensitivity analysis of associations between candidate risk factors and distant metastasis. **Table S3.** Distributions of candidate risk factors according to recurrence types. **Table S4.** Summarized effect estimates of multivariable Cox regression in discovery analysis.

## Data Availability

All data supporting this study are available from the corresponding author upon reasonable request.

## Abbreviations

CRC: Colorectal cancer
DFS: Disease-free survival
OS: Overall survival
HR: Hazard ratio
CEA: Carcinoma embryonic antigen
PNI: Perineural invasion
ESMO: European Society of Medical Oncology
NCCN: National Comprehensive Cancer Network
WCH: West China Hospital
PUCHI: Peking University Cancer Hospital & Institute
AJCC: American Joint Committee on Cancer
LVI: Lymphovascular invasion
CI: Confidence interval
AUC: Area under the curve
DCA: Decision curve analysis
RCS: Restricted cubic splines
CSS: CRC-specific survival
