# Supplementary material for: Survival outcomes of stage I colorectal cancer: development and validation of the ACEPLY model using two prospective cohorts
Source: BMC Med. 2023 Jan 4;21:3. doi: 10.1186/s12916-022-02693-7 (PMC9814451; doi:10.1186/s12916-022-02693-7)
Supplement: Supplementary file 2 — Additional file 2. Follow-up scheme for patients. [file 12916_2022_2693_MOESM2_ESM.pdf]

Additional file 2: Follow-up scheme for patients

Patients were referred to the follow-up clinic every three months during the first two years after surgery, then every six months from the third to the fifth year, and every 12 months after five years from the date of surgery. For each visit, patients undertook physical examination, blood cell test, tumor biomarkers including CEA levels. Computed tomography (CT) of the chest and CT or magnetic resonance imaging (MRI) of the abdominal and pelvic region were employed to identity any possible recurrences every six months within the first two years and every 12 months from then on. Colonoscopies were also performed every two years after the surgery for each participant.
