# Supplementary material for: Survival outcomes of stage I colorectal cancer: development and validation of the ACEPLY model using two prospective cohorts
Source: BMC Med. 2023 Jan 4;21:3. doi: 10.1186/s12916-022-02693-7 (PMC9814451; doi:10.1186/s12916-022-02693-7)
Supplement: Supplementary file 3 — Additional file 3: Figure S1-S3. Figure S1. Kaplan-Meier curves of DFS (A), OS (B) and recurrence rate (C) for the two study cohorts. Figure S2. Performance of the multivariable prediction model on OS. Figure S3. Performance of the multivariable prediction model on recurrence rate. [file 12916_2022_2693_MOESM3_ESM.pdf]

Additional file 3: Figure S1-S3.

Figure S1: Kaplan-Meier curve of DFS (A), OS (B) and recurrence rate (C) for the two study cohorts

Figure S2: Performance of the multivariable prediction model on OS

Figure S3: Performance of the multivariable prediction model on recurrence rate

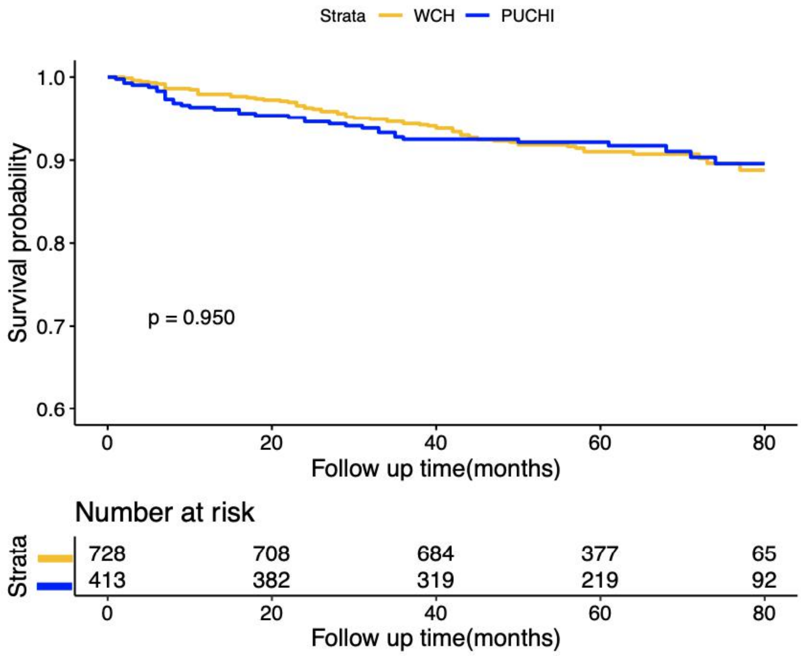

Figure S1. Kaplan-Meier curve of DFS (A), OS(B) and recurrence rate (C) for the two study cohorts

A

B

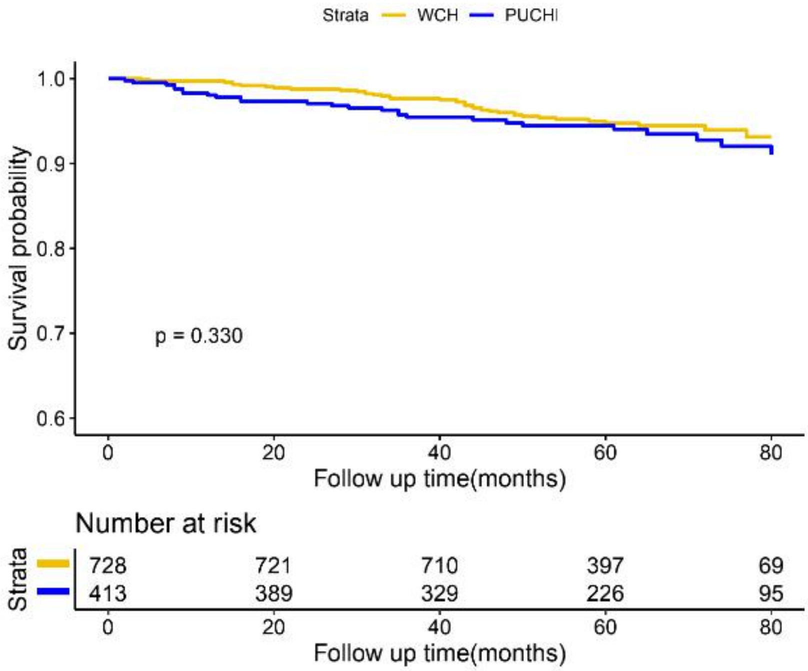

C

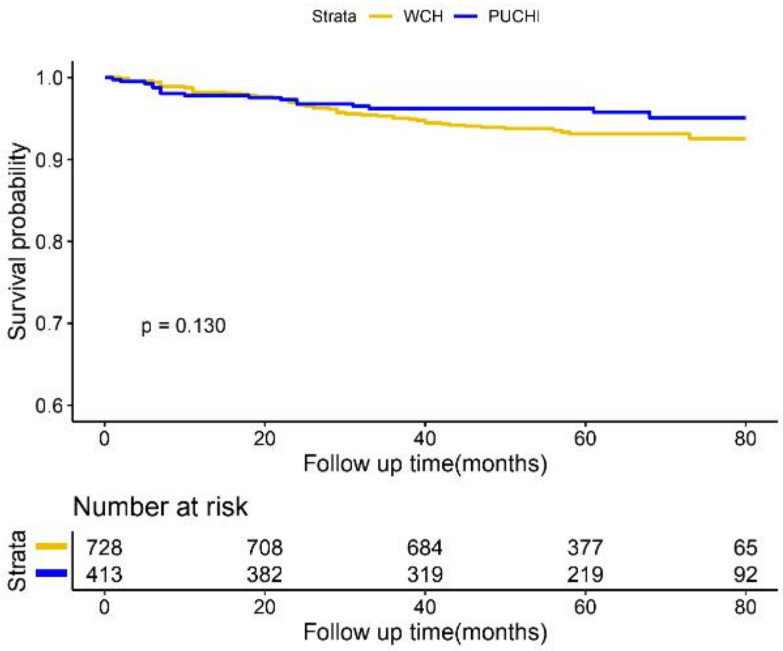

Strata
WCH
PUCHI
1.0
0.9
0.8
0.7
0.6
Survival probability
p = 0.130
0
20
40
60
80
Follow up time(months)
Number at risk
Strata
728
708
684
377
65
413
382
319
219
92
0
20
40
60
80
Follow up time(months)

Figure S2. Performance of the multivariable prediction model on OS

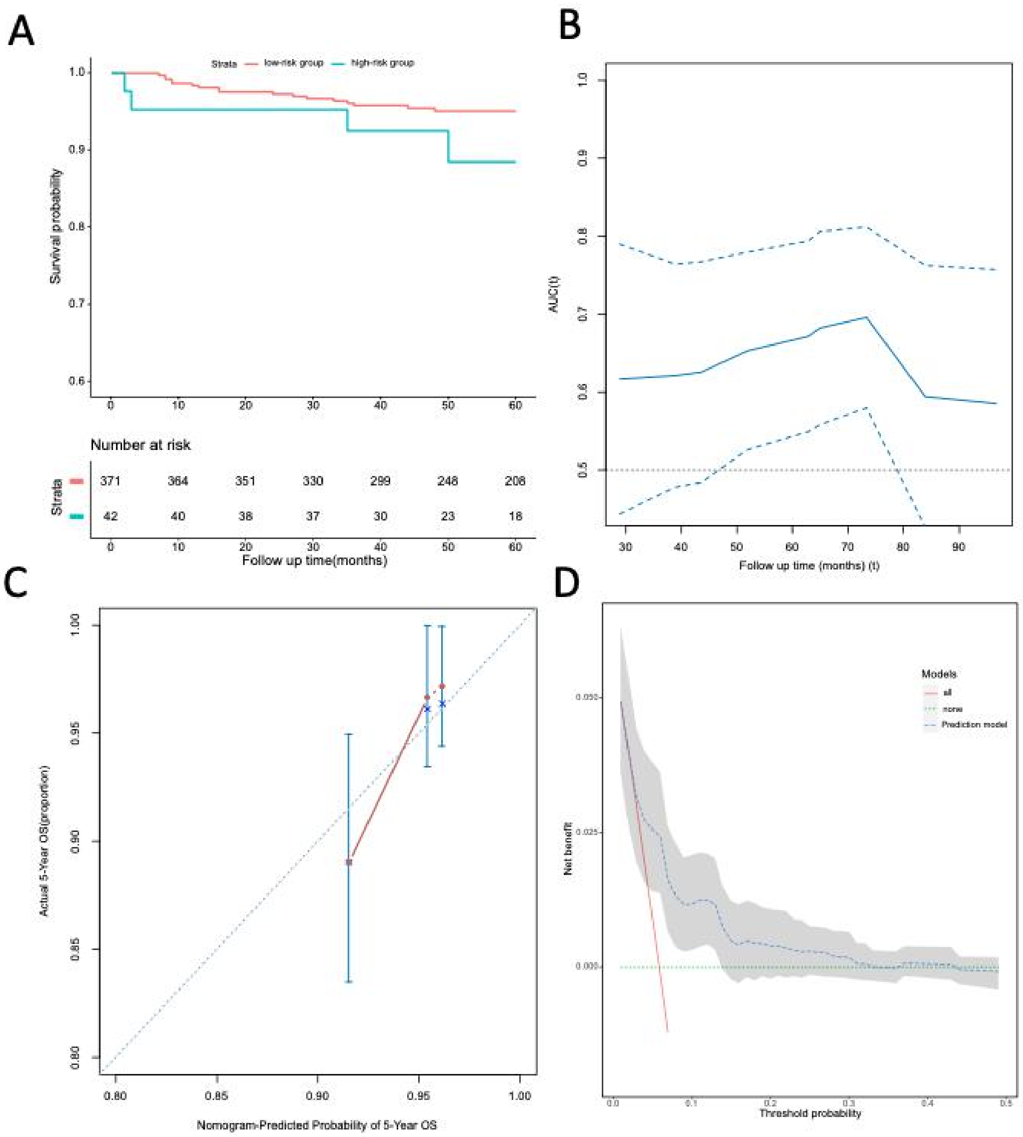

A.Kaplan-Meier curve of high- and low-risk group of stage I patients in the validation cohort based on linear prognostic index with a cut-off value derived from the discovery cohort. B. Time-dependent area under the curve (AUC) of the prediction model validated in the external cohort. C.Model calibration in the validation cohort. D.Decision curve analysis of the prediction model. The probability threshold indicates the ratio of benefit of true positives vs. the harm of false positives.

Figure S3. Performance of the multivariable prediction model on recurrence rate

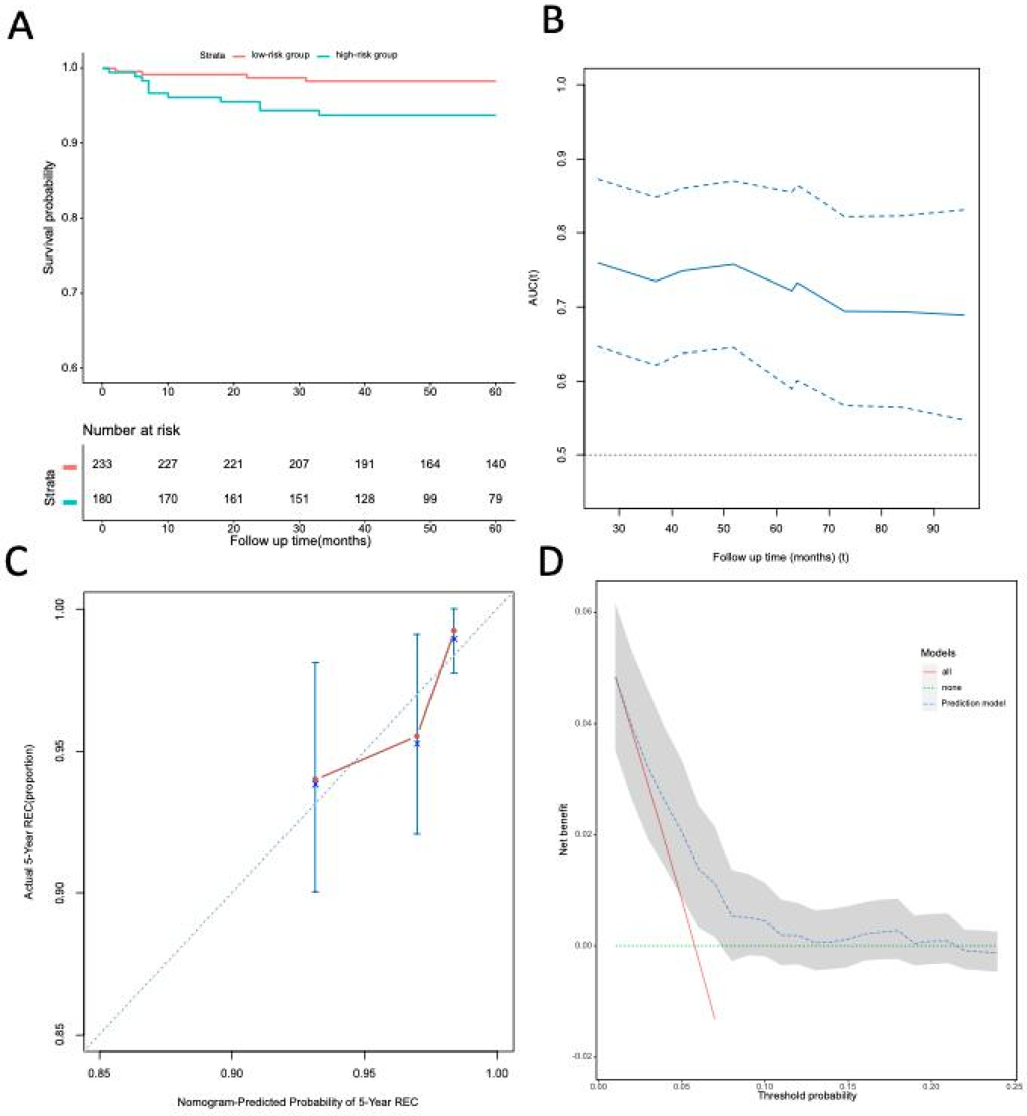

A.Kaplan-Meier curve of high- and low-risk group of stage I patients in the validation cohort based on linear prognostic index with a cut-off value derived from the discovery cohort. B. Time-dependent area under the curve (AUC) of the prediction model validated in the external cohort. C.Model calibration in the validation cohort. D.Decision curve analysis of the prediction model. The probability threshold indicates the ratio of benefit of true positives vs. the harm of false positives.
