# Supplementary material for: Survival outcomes of stage I colorectal cancer: development and validation of the ACEPLY model using two prospective cohorts
Source: BMC Med. 2023 Jan 4;21:3. doi: 10.1186/s12916-022-02693-7 (PMC9814451; doi:10.1186/s12916-022-02693-7)
Supplement: Supplementary file 4 — Additional file 4: Table S1-S4. Table S1. Summarized results of sensitivity analysis of associations between candidate risk factors and CRC-specific survival. Table S2. Summarized results of sensitivity analysis of associations between candidate risk factors and distant metastasis. Table S3. Distributions of candidate risk factors according to recurrence types. Table S4. Summarized effect estimates of multivariable Cox regression in discovery analysis. [file 12916_2022_2693_MOESM4_ESM.pdf]

Additional file 4: Table S1-S4

Table S1: Summarized results of sensitivity analysis of associations between candidate risk factors and CRC-specific survival

Table S2: Summarized results of sensitivity analysis of associations between candidate risk factors and distant metastasis

Table S3: Distributions of candidate risk factors according to recurrence types

Table S4: Summarized effect estimates of multivariable Cox regression in discovery analysis

Table S1. Summarized results of sensitivity analysis of associations between candidate risk factors and CRC-specific survival

Factors	Discovery analysis			Validation analysis		
	HR	95% CI	P-value	HR	95% CI	P-value
Age	**1.05**	**(1.01, 1.10)**	**0.013**	1.04	(0.97, 1.11)	0.292
Gender (Male)	1.31	(0.54, 3.17)	0.546		——	
Grade (G3 or 4)	1.64	(0.64, 4.23)	0.305		——	
CEA*	**1.91**	**(1.31, 2.79)**	**<0.001**	**2.47**	**(1.17, 5.21)**	**0.018**
LVI	NA				——	
Lymph node retrieval (<12)	2.22	(0.89, 5.49)	0.085		——	
PNI	**8.56**	**(2.50, 29.36)**	**<0.001**	NA		
T stage (T2)	6.17	(0.83, 46.00)	0.076		——	

NA, not available given no events presented in the risk group. CEA, carcinoma embryonic antigen; LVI, lymphovacular invasion, PNI, perineural invasion

*CEA levels were log-transformed

Table S2. Distributions of candidate risk factors according to recurrence types

	Recurrence site		P-value
Risk factors	Local (N=9)	Distant (N=57)	
Tumor grade			0.19
G1 or G2	9(100%)	43(75.4%)	
G3 or G4	0(0%)	14(24.6%)	
T stage			0.17
T1	3(33.3%)	8(14.0%)	
T2	6(66.7%)	49(86.0%)	
Lymph node retrieval			0.45
<12	5(55.6%)	40(70.2%)	
≥12	4(44.4%)	17(29.8%)	
LVI			0.53
Yes	1(11.1%)	4(7.0%)	
No	8(88.9%)	53(93.0%)	
PNI			
Yes	1(11.1%)	6(10.5%)	0.99
No	8(88.9%)	51(89.5%)	
CEA*	1.13(0.12-1.44)	1.11(0.75-1.67)	0.38

CEA, carcinoma embryonic antigen; LVI, lymphovacular invasion, PNI, perineural invasion

*CEA levels were log-transformed

Table S3. Summarized results of sensitivity analysis of associations between candidate risk factors and distant recurrences

Factors	Discovery analysis			Validation analysis		
	HR	95% CI	P-value	HR	95% CI	P-value
Age	1.02	(0.99, 1.05)	0.170		——	
Gender (Male)	1.54	(0.83, 2.88)	0.173		——	
Grade (G3 or 4)	1.21	(0.60, 2.45)	0.594		——	
CEA*	**1.58**	**(1.18, 2.13)**	**0.002**	1.74	(0.94, 3.22)	0.079
LVI	1.83	(0.57,5.91)	0.313		——	
Lymph node retrieval (<12)	**2.66**	**(1.39, 5.08)**	**0.003**	1.71	(0.57,5.08)	0.338
PNI	**4.99**	**(1.77, 14.01)**	**0.002**	**6.46**	**(1.40,29.82)**	**0.017**
T stage (T2)	**3.08**	**(1.10, 8.60)**	**0.032**	0.73	(0.23,2.37)	0.602

NA, not available given no events presented in the risk group. CEA, carcinoma embryonic antigen; LVI, lymphovacular invasion, PNI, perineural invasion

*CEA levels were log-transformed

Table S4: Summarized effect estimates of multivariable Cox regression in discovery analysis

	**Disease-free survival**			**Overall survival**			**Recurrence**		
Risk factors	HR	95%CI	P	HR	95%CI	P	HR	95%CI	P
Age	**1.03**	**(1.01,1.06)**	**0.007**	**1.09**	**(1.06,1.13)**	**<0.001**		——	
CEA*	**1.34**	**(1.01,1.78)**	**0.041**	1.37	(0.95,1.97)	0.089	**1.58**	**(1.17,2.14)**	**0.003**
PNI	**3.36**	**(1.34,8.46)**	**0.010**	**4.45**	**(1.55,12.76)**	**0.006**	**3.96**	**(1.42,11.08)**	**0.009**
Node retrieval (<12)	**1.85**	**(1.11,3.08)**	**0.019**		——**		**2.40**	**(1.32,4.37)**	**0.004**
T stage (T2)	1.85	(0.87,3.94)	0.112	2.40	(0.72,7.96)	0.153		——	

CEA: Carcinoma Embryonic Antigen; PNI: perineural invasion;

*CEA levels were log-transformed

**Variables were not included given non-significant findings in univariable analysis
